# Re-localization of hormone effectors is associated with dormancy alleviation by temperature and after-ripening in sunflower seeds

**DOI:** 10.1038/s41598-019-40494-w

**Published:** 2019-03-19

**Authors:** Qiong Xia, Maharajah Ponnaiah, Kaviya Thanikathansubramanian, Françoise Corbineau, Christophe Bailly, Eiji Nambara, Patrice Meimoun, Hayat El-Maarouf-Bouteau

**Affiliations:** 1 Sorbonne Université, CNRS, Biologie du développement Paris Seine - Institut de Biologie Paris Seine, LBD - IBPS, 75005 Paris, France; 2 Department of Cell and Systems Biology, University of Toronto, Toronto, ON M5S 3B2 Canada

## Abstract

Temperature is the primary factor that affects seed dormancy and germination. However, the molecular mechanism that underlies its effect on dormancy alleviation remained largely unknown. In this study, we investigate hormone involvement in temperature induced germination as compared to that caused by after-ripening. Dormant (D) sunflower seeds cannot germinate at 10 °C but fully germinate at 20 °C. After-ripened seeds become non-dormant (ND), i.e. able to germinate at 10 °C. Pharmacological experiments showed the importance of abscisic acid (ABA), gibberellins (GAs) and ethylene in temperature- and after-ripening-induced germination of sunflower seeds. Hormone quantification showed that after-ripening is mediated by a decline in both ABA content and sensitivity while ABA content is increased in D seeds treated at 10 or 20 °C, suggesting that ABA decrease is not a prerequisite for temperature induced dormancy alleviation. GAs and ethylene contents were in accordance with germination potential of the three conditions (GA_1_ was higher in D 20 °C and ND 10 °C than in D 10 °C). Transcripts analysis showed that the major change concerns ABA and GAs metabolism genes, while ABA signalling gene expression was significantly unchanged. Moreover, another level of hormonal regulation at the subcellular localization has been revealed by immunocytolocalization study. Indeed, ABA, protein Abscisic acid-Insensitive 5 (ABI5), involved in ABA-regulated gene expression and DELLA protein RGL2, a repressor of the gibberellins signalling pathway, localized mainly in the nucleus in non-germinating seeds while they localized in the cytosol in germinating seeds. Furthermore, ACC-oxidase (ACO) protein, the key ethylene biosynthesis enzyme, was detected in the meristem only in germinating seeds. Our results reveal the importance of hormone actors trafficking in the cell and their regulation in specialized tissue such as the meristem in dormancy alleviation and germination.

## Introduction

Seed dormancy and germination are complex adaptive traits of higher plants, they are determined by a combination of the degree of dormancy and environmental factors such as temperature, light, and oxygen^1,2^. Temperature is a primary factor regulating seed dormancy and germination. At harvest, dry seeds are dormant and may experience gradual dormancy loss through dry after-ripening. After-ripening is a time and environment regulated process occurring in the dry seed. The process by which dormant seeds become non-dormant determines the germination potential of seeds^3,4^. As dormancy reduces the range of external conditions under which germination can occur, removal of dormancy by after-ripening enlarges the temperature range under which seeds can germinate. The freshly harvested mature seeds of sunflower (*Helianthus annuus* L.) are regarded as being deeply dormant because they germinate poorly at 10 °C or lower temperatures, they are able to germinate at temperatures ranging from 20 to 30 °C. Few months of after-ripening by dry storage breaks their dormancy, sunflower seeds become able to germinate at temperatures ranging from 5 to 40 °C, and their germination rate is enhanced at all temperatures^5^.

The mechanism of endogenous plant hormonal regulation is supposed to be highly conserved in seed dormancy and germination processes. Many of the environmental controlled responses seem to be mediated via the regulation of hormonal content and/or signal transduction^6^. Genetic and physiological studies have shown the important roles of the plant hormones, abscisic acid (ABA) and gibberellins (GAs) in seed dormancy. Other hormones such as ethylene and brassinosteroids, which both influence the ABA/GAs balance by counteracting ABA effects, promote germination^7^. ABA is a positive regulator of dormancy while GAs and ethylene release dormancy and promote the completion of germination by counteracting the effects of ABA^2^. Alteration in the ABA biosynthetic pathway can greatly influence seed dormancy and germination^8–10^. Decline in ABA content, decreased sensitivity to ABA and increased sensitivity to GAs are involved in the after-ripening mediated transition from the dormant to the non-dormant state of many species^11–14^. Moreover, exogenous GAs supply can substitute for the after-ripening requirement in many species and after-ripening mediated dormancy release is correlated with GAs requirement^15,16^. Similarly, ethylene and its immediate precursor (1-aminocyclopropane-1-carboxylic acid, (ACC)) completely break seed dormancy and improve seed germination in several species^17^.

Several key genes/enzymes have been characterized in ABA biosynthesis and catabolism^10^. In Arabidopsis, mutations of ABA biosynthetic genes, including ZEP/ABA1^18^, NCEDs^19,20^ and ABA2/GIN1/SDR1^21,22^, result in reduced dormancy, whereas over-expression of ABA biosynthetic enzymes enhances dormancy^9,23^. Mutant of the CYP707A2, the gene that encodes the enzyme catalyzing ABA hydroxylation, presents a strong seed dormancy phenotype^24,25^. By contrast, loss of function in gibberellins biosynthetic genes such as *ent*-copalyldiphosphate synthase (CPS/GA1), *ent*-kaurene synthase (KS/GA2), *ent*-kaurene oxidase (KO/GA3) and gibberellin-3-oxidases (GA3ox1 and GA3ox2) caused to fail germination^26,27^. For ethylene, it was demonstrated that ACC oxidase (ACO) activity plays a fundamental role in the promotion of seed germination^28,29^.

The signaling pathways of ABA and GAs and ethylene are interconnected at several levels. A basic domain/leucine zipper transcription factor ABI5 (ABA-INSENSITIVE 5) acts as key regulator in ABA signalling, while DELLA protein RGL2 (RGA-LIKE2), which repress germination, is considered to be the main negative regulating factor of GAs signalling^30,31^. ABA induces ABI5 expression to repress germination, and GAs promotes seed germination by enhancing the proteasome-mediated destruction of RGL2. Furthermore, several ethylene mutants affected in ethylene metabolism or signaling, present differential sensitivity to ABA^32^.

Although the phytohormones involved in dormancy and germination have been largely identified, their mechanisms of interaction with external factors and how dormancy is broken under different conditions are more elusive. The links between temperature and hormones have been shown only in germination thermoinhibition process^33^. A decrease in ABA content is suppressed and *de novo* ABA biosynthesis is required during lettuce seed imbibition at supra-optimal temperature^34^. This thermoinhibition can be alleviated by application of ABA biosynthesis inhibitor like fluridone^35–37^. Since GAs are generally required for seed germination, alleviation of thermoinhibition by exogenous GAs has been reported for several plant species^36,38,39^. In Arabidopsis, the expression of GAs biosynthetic genes GA3ox and GA20ox can be suppressed by thermoinhibition while they were induced by lower temperatures^27,37^. On the other hand, it is also known that application of ethylene or ACC can release the thermoinhibition in lettuce, chickpea, sunflower and tomato seeds^39–42^. All of these reports suggest that ABA, GAs and ethylene are involved in the regulation of seed germination by temperature, but how these hormones mediate the optimal temperature signal during dormancy release remains unknown.

Furthermore, currently, increasing interest is given to transport of hormones from/to the different tissues of the seed and consequently their contribution in each tissue as it appears to be a critical factor regulating hormone responses in the seed^43^. Furthermore, the integration of such multiple inputs by mapping the different hormones at the same time in the different seed tissue and subcellular level can be determinant in the understanding of germination process.

This paper describes dormancy regulation by temperature or after-ripening during the germination *sensu stricto* using two imbibition times in the phase I, characterised by rapid water uptake (3 and 6 h) and 2 imbibition times in the phase II, characterised by the arrest of water uptake before radicle protrusion (15 and 24 h). The effects of ABA, GAs and ethylene during seed dormancy release in sunflower by temperature as compared to after-ripening have been investigated by hormone quantification, gene expression analysis of key actors in hormone metabolism and signaling and spatial localization of hormone action in sunflower embryos.

## Material and Methods

### Plant material

Sunflower (*Helianthus annuus* L. cv LG5662) seeds were produced in open fields in the south of France by Valgrain (26740 Les Tourrettes, France) in 2014. Dormant seeds were stored at −20 °C until use to maintain their dormancy after harvest, and non-dormant seeds were obtained after dormancy release at 20 °C and 75% relative humidity during 3 months.

### Germination tests and hormones treatment

Germination tests and hormone treatments were performed with naked seeds (seeds without pericarp) imbibed on a layer of cotton wool moistened with deionised water or with various solutions (100 µM ABA, 10 µM fluridone, 100 µM GA_3_, 100 µM paclobutrazol (PAC), 1 mM aminooxyacetic acid (AOA), and 1 mM α-aminoisobutyric acid (AIB)) in 9 cm Petri dishes under darkness at 10 °C or 20 °C. Treatment with ethylene was carried out by placing Petri dishes in a tightly closed glass jar continuously in the presence of 100 ppm gaseous ethylene. An embryo was considered as germinated when the radicle had elongated up to 2 mm. The results presented were obtained with 25 seeds per dish and six replicates.

### Quantification of GAs and ABA

GAs and ABA were extracted from 100 mg dry weight (DW) of embryonic axes of imbibed seeds. The purification and measurement were performed by LC-ESI-MS/MS based analysis as described in Yano, *et al*.^44^ with some modifications. Briefly, seeds were frozen with liquid nitrogen, homogenised, freeze dried and stored at −80 °C until use. Stable isotope labeled standards,D_6_-ABA, D_2_-GA_1_, D_2_-GA_20_ (Olchemim), were added, and hormones were extracted three times with 1 ml of 80% (v/v) methanol containing 1% (v/v) acetic acid, and dried up *in vacuum*. Solid phase extraction with three different disposable cartridge columns (Waters), Oasis HLB, WCX, and WAX, were used to purify hormonal fractions as described in Yano, *et al*.^44^. Subsequently, resultant samples were subjected to two rounds of HPLC fractionations with a C18 column. The first round of fractionation was performed with mobile phase as water containing 1% (v/v) acetic acid: methanol containing 1% (v/v) acetic acid, while the second round as water containing 1% (v/v) acetic acid:acetonitrile. The samples were subjected to an LC-ESI-MS/MS (Agilent) equipped with a phenyl column. Results are expressed as ng/g DW and represent the mean of three replicates ± SD.

### Quantification of ethylene

To measure the ethylene production, 5 naked seeds were placed in 10 ml flasks. Flasks were tightly closed with serum caps and placed under darkness at 10 °C or 20 °C. One ml gas sample was taken from each flask and injected into a gas chromatograph (Hewlett Packard 5890 series II) equipped with a flame ionisation detector and an activated alumina column for ethylene determination using ethylene gas as internal standard. Results are the means of five measurements ± SD and are expressed as nl ethylene h^−1^ per seed.

### Total RNA extraction

Total RNA was extracted by an improved procedure according to Oñate-Sánchez, *et al*.^45^. Twenty isolated embryonic axes were ground to a fine powder in liquid nitrogen, and then transferred to a cooled 2 ml tube containing 550 µl of extraction buffer (0.4 M LiCl, 0.2 M Tris pH 8, 25 mM EDTA, 1% (v/v) SDS) and 550 µl of chloroform. Suspensions were vortexed for 2 min and centrifuged for 5 min at 13000 rpm. The supernatant was transferred to a new 2 ml tube and 500 µl of phenol were added. After using vortex thoroughly, 200 µl of chloroform/CIA 24:1 was added and extract was centrifuged for 5 min at 13000 rpm. One volume of 4 M LiCl was then added to the supernatant and mixed. After overnight precipitation at 4 °C and centrifugation for 45 min at 13000 rpm, the pellet was dissolved in 250 µl Milli-Q water and precipitated once again with 25 µl 2.5 M NaAc and 500 µl ethanol. After washing the pellet with 70% EtOH three times, RNA was resuspended in 100 µl Milli-Q water after air-dry.

### Design of primers and real-time qRT-PCR analysis

The oligonucleotide primer sets used for real-time qPCR analysis were designed on the basis of sunflower gene sequences (https://www.heliagene.org). All the genes used and the related heliagene accession number, amplified probe length and primer sequences are listed in S1 Table. ACO primers correspond to those used in Oracz, *et al*.^46^.

Two µg total RNA was reverse-transcribed and amplified, and the relative expression was calculated with three reference genes as described by Meimoun, *et al*.^47^. Gene expression analyses were performed on seeds from 3 different lots. The distance matrix expression analysis was performed by sorting points into neighbourhoods (SPIN) algorithm by Euclidian distance^48^. The PCA analysis was carried-out using Clustvis tool^49^.

### Immunolocalization of ABA, ABI5, RGL2, and ACO

For immunolocalization studies, sections at a thickness of 25 μm were obtained by using vibratome (LEICA, VT 1200S) from imbibed seeds for 15 h, and then were fixed overnight at 8% (w/v) paraformaldehyde in 0.1 M PBS (pH 7.4). To allow fixation of ABA *in situ*^50^, all sections were fixed for 24 h with 3% (w/v) paraformaldehyde in 4% (w/v) 1-ethyl-3-(3-dimethylaminopropyl) carbodiimide (Sigma-Aldrich Co., St Louis, MO, USA) diluted in PBS at 4 °C. After fixation step, the sections were transferred to the normal goat serum diluted to 1:30 in PBST (PBS containing 0.1% (v/v) of Tween 20) for 20 min. They were washed in PBST and incubated overnight at 4 °C with the 1:200 dilution primary antibodies anti-ABI5; anti-RGL2 and anti-ACO^51,52^. ABA antibody used for immunolocalization in roots^50^ has been tested in seeds comparing Arabidopsis wild type dormant and non-dormant with hyperdormant *cyp707a2* mutant seeds accumulating high content of ABA^24^ (Fig. S1). After washing three times in PBST for 10 min, sections were incubated with Alexa 488 conjugated anti-rabbit secondary antibody (Molecular Probes, Invitrogen, Germany) for 1 h in darkness. Then, the sections were washed three times for 10 min in PBST and were observed with a fluorescence Zeiss microscope (excitation filter 450–490 nm). Nuclei were visualised with DAPI (300 nM) co-staining.

### Statistical analysis

Differences between treatments were determined by the Kruskal-Wallis test by using XLSTAT software, and were considered significant when p-values < 0.05.

## Results

### Seed responsiveness to exogenous GAs, ABA, ethylene and their inhibitors upon dormancy release by temperature or after-ripening treatments

**Figure 1:**
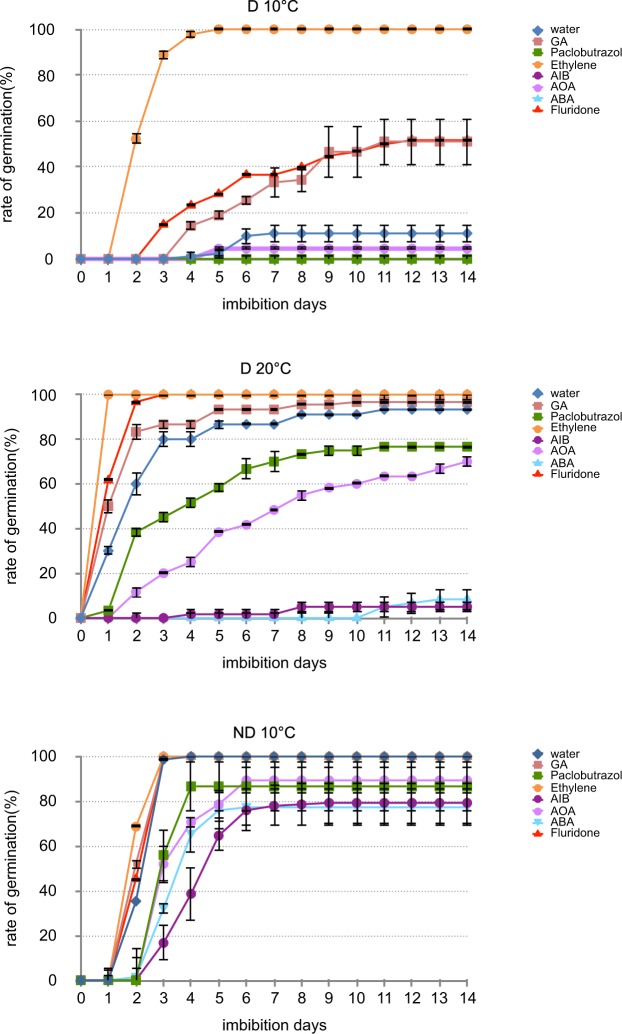
Germination of naked sunflower seeds at 10 °C or 20 °C on water or in presence of different treatments. Dormant seeds were incubated at 10 °C (D 10 °C) or 20 °C (D 20 °C) and non-dormant seeds were incubated at 10 °C (ND 10 °C), in presence of ABA (100 µM) and its inhibitor fluridone(10 µM), GA (100 µM) and its inhibitor paclobutrazol (PAC, 100 µM), gaseous ethylene (100ppm) and its inhibitors, aminooxyacetic acid (AOA, 1 mM) and α-aminoisobutyric acid (AIB, 1 mM). Values are means of three replicates ± standard deviation.

We analysed germination responses upon treatments with exogenous GA_3_, ABA and ethylene (ET) and some of their biosynthesis inhibitors, paclobutrazol (PAC), fluridone and AIB, respectively. As shown in Fig. 1, dormant seeds are sensitive to exogenous ABA as germination percentage at 10 and 20 °C was strongly reduced from 11 to 0% and 93 to 8%, respectively. ND seeds seemed to be less sensitive to ABA treatment but germination percentage was reduced (Fig. 1). Accordingly, the ABA synthesis inhibitor fluridone increased the germination of dormant seeds at 10 and 20 °C and had no effect on ND seeds already fully germinating. As observed with fluridone, addition of GA_3_ increased dormant seeds germination percentage from 10 to 51.7% at 10 °C. GA synthesis inhibition with PAC reduced the germination of seeds from the different treatments but more effect was observed for D 20 °C (Fig. 1). Addition of ET induced a complete dormancy release at 10 °C and ethylene synthesis inhibitors AOA and AIB, significantly prevent seed germination at 20 °C when the effect on ND seeds was less important (Fig. 1). As a whole these data confirmed the role of ABA in maintaining dormancy and the role of GAs and ET in alleviation of dormancy. Moreover, D seeds are more sensitive to hormones than ND ones.

### Temperature and after-ripening effects on hormone contents

**Figure 2:**
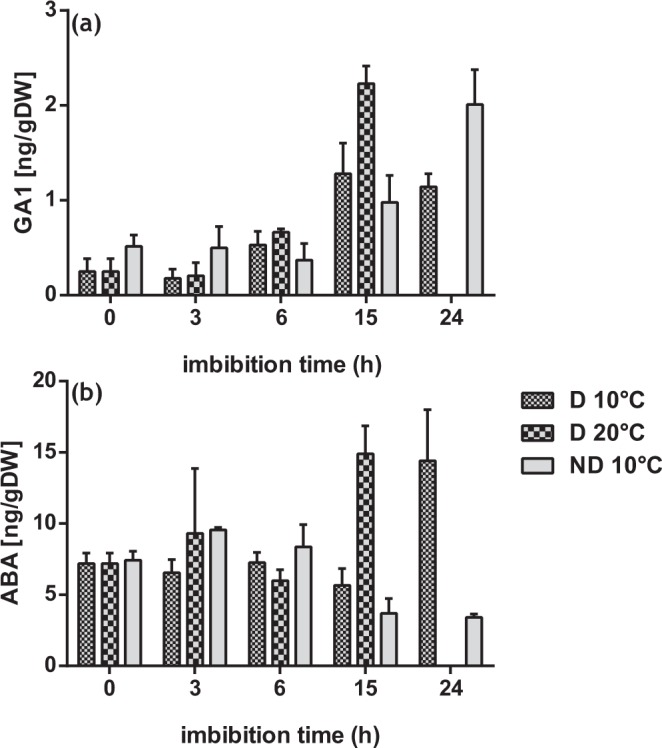
GA_1_ and ABA contents in dormant (D) and non-dormant (ND) axes isolated from sunflower seeds incubated on water at 10 °C and 20 °C. (**a**) Endogenous content of bioactive GA1; (**b**) Endogenous content of ABA. Values are means of three replicates ± standard deviation. At 24 h, hormone contents were not analyzed for D 20 °C as 30% of the seed population has germinated (Fig. 1). Differences between treatments were determined by the Kruskal-Wallis test (p-values < 0.05).

We thus explored the real changes in endogenous hormone levels during temperature-induced dormancy alleviation and after-ripening. ABA, GA1 and ET were quantified in axes isolated from D 10 °C, ND 10 °C and D 20 °C. Five imbibition time points were chosen for quantification, 0, 3, 6, 15 and 24 h. GA1 is the main active GAs component in sunflower embryo axes, it started to increase in D seeds since 6 h to reach high value at 15 h for D 20 °C (p-value < 0.0001) comparable to that of ND 10 °C at 24 h (p-value < 0.0001) (Fig. 2a). It is important to note that GA1 content was largely higher in germinating seeds with a time lag which can be explained by the fact that D 20 °C germinate faster than ND 10 °C. Consequently, because of the high germination percentage of seeds corresponding to D 20 °C at 24 h, hormones have not been quantified from these seeds at this point (Fig. 2). ABA content was about 7.2 to 7.5 ng g−1 DW in the embryos of dry seeds. Slight increase has been recorded in ND 10 °C after 3 h of imbibition (Fig. 2b). ABA content increased at 24 h for D 10 °C (p-value < 0.0001) while it decreased for ND seeds since 15 h (p-value = 0.000108) (Fig. 2b). Surprisingly, ABA content increased markedly at 15 h of imbibition for D 20 °C (p-value < 0.0001) even the seeds can germinate. Ethylene measurement did not allow detecting ET in any seed condition (not shown). These results indicated that GA content correlates with seed germination potential but not ABA and ET contents, indicating the importance in considering the main hormones together in each condition.

### Temperature and after-ripening affect gene expression of hormone metabolism and signalling related genes

**Figure 3:**
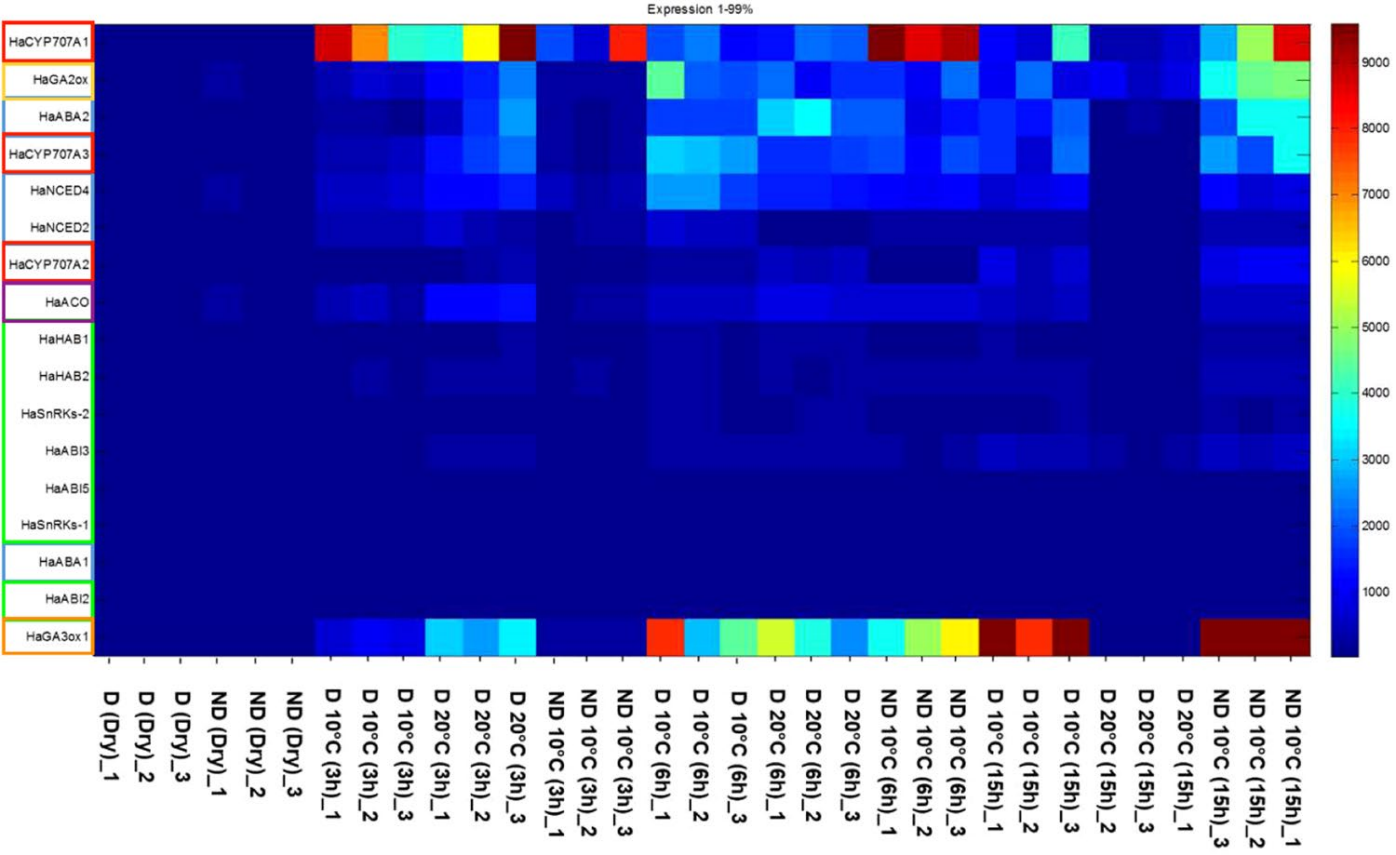
Heatmap depicting expression of genes involved in the hormone (ABA, GAs and ethylene) metabolism and (ABA) signalling. Dormant (D) and non-dormant (ND) sunflower embryos at dry state (dry) or during imbibition (3, 6 or 15 h) at 10 °C or 20 °C in three replicates (−1, −2 and −3).

To go further in the molecular basis of hormone involvement in temperature as compared to after-ripening regulation of germination, we investigated the gene expression of key actors of hormone metabolism and signalling. Four ABA biosynthesis related genes (*HaABA1*, *HaABA2*, *HaNCED2* and *HaNCED4*) have been analyzed during imbibition of D 10 °C, D 20 °C and ND 10 °C (Fig. 3). *HaABA1* gene expression did not show significant change in any condition. *HaNCED2* showed increased more importantly in D seeds especially at 3 and 6 h (Fig. 3). The most drastic increase in gene expression concerns *HaNCED4* and *HaABA2*, more importantly in D 10 and 20 °C at 3 and 6 h (Fig. 3). Concerning ABA oxidation related genes, *HaCYP707A2* showed slight change comparing to *HaCYP707A1* and *HaCYP707A3* (Fig. 3). *HaCYP707A1* was up-regulated since 3 h in all conditions to reach high level in ND 10 °C especially at 6 h. The expression of seven genes (*HaSnRKs1*, *HaSnRKs2*, *HaHAB1*, *HaHAB2*, *HaABI2*, *HaABI3*, *HaABI5*) involved in ABA signalling pathway have also been analyzed (Fig. 3). Their corresponding gene expression level was too low compared to that of metabolism related genes without marked change between conditions or imbibition times for *HaABI2 and HaABI5* and slight increase in D compared to ND seeds at 3 and 6 h for *HaSnRKs1*, *HaSnRKs2*, *HaHAB1*, *HaHAB2 and HaABI3* (Fig. 3). *HaGA3ox1* and *HaGA2ox*, corresponding to GA3-oxidases and GA2-oxidases involved in synthesis and degradation of bioactive GAs, respectively, both showed an increased gene expression in the different conditions during imbibition (Fig. 3). *HaGA2ox* increased first in D seeds (at 3 h), then it increased gradually at 6 and 15 h in D 10 °C and in ND 10 °C. The same pattern has been recorded for *HaGA3ox1*. The *HaACO* gene expression increased more importantly in D 20 °C at 3 h and then decreased while it increased in D and ND at 6 and 15 h (Fig. 3).

For all the analyzed genes, our results showed that at dry state there is no significant change in gene expression between D and ND. Moreover, all these hormone-related genes were down-regulated in D 20 °C at 15 h which correspond to the end of the phase II of the germination *sensu stricto* in this condition. Indeed, germination of D at 20 °C was faster than ND at 10 °C as increased temperature can break dormancy but also increase the rate of germination. This is in accordance with the different GA levels recorded in D 20 °C and ND between 15 and 24 h (Fig. 2a).

**Figure 4:**
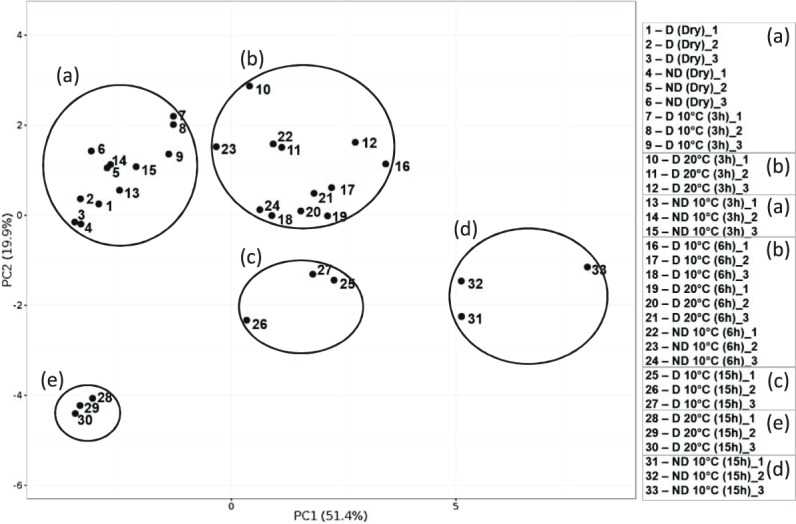
Principal components analysis considering all gene expression data set. PC1 = 51,4%, PC2 = 19,9%. Five groups have been clustered according to their distance based on the two axes (**a**–**e**). The plots were constructed using the Clustvis tool.

To examine differences and similarities between conditions, PCA analysis was performed. Samples have been clustered in five groups ((a), (b), (c), (d) and (e)) according to their distance based on the PCA axes, PC1 (51,4%) and PC2 (19,9%, Fig. 4). Dry and 3 h imbibed D and ND at 10 °C were clustered in the same group (a). All samples corresponding to 6 h of imbibition were clustered with D 20 °C 3 h in the group (b). D 10 °C imbibed for 15 h was differentiated from the (b) group only by the PC2 ((c) group), while there was a clear shift of ND 10 °C 15 h on the PC1 ((d), Fig. 4). D 20 °C (15 h) was different by PC2 and similar by the PC1 to the (a) group. All these results suggest that the time course of gene expression is important in hormone interaction and subsequent signalling and highlight the different time of regulation in hormonal balance between D 20 °C and ND to allow germination. To further explore if there is also a spacial control of hormone actions, we investigated the immunolocalization of ABA, GA and ethylene related proteins.

### Immunolocalization of ABI5, RGL2, and ACO

**Figure 5:**
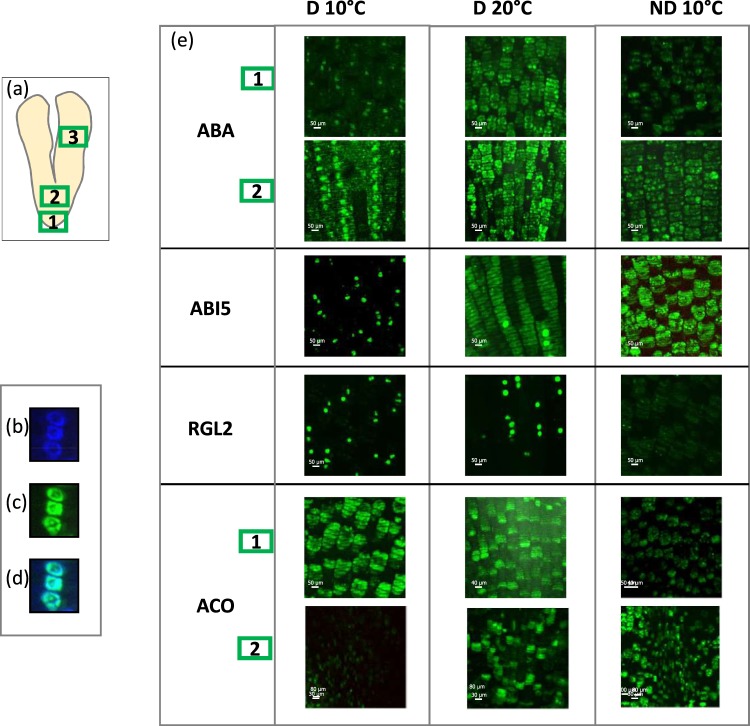
Immunolocalization of ABA, ABI5, RGL2 and ACO in sunflower seeds. Longitudinal sections were prepared from naked seeds imbibed after 15 h on water. All the different parts of the embryo were observed and representative pictures are shown. (**a**) Schematic longitudinal section of the embryo representing the different parts, 1, axis, 2, meristem, 3, cotyledon. Nuclei labelling controls:(**b**) blue label showing the nuclei that were stained with DAPI (4′,6-diamidino-2-phenylindole), green label indicates the distribution of ABI5 in the axis (**c**), and merged image (**d**). (**e**) Localization of ABA, ABI5, RGL2 and ACO. Green label indicates the interaction of different antibodies with their antigen.

A spatial localization of hormone effectors was studied by immunocytolocalization, using antibodies anti-ABA, -ABI5, -RGL2 and -ACO as probes. The whole embryo has been observed with particular attention to important histological zones, such as axis, meristem or cotyledon (Fig. 5a). As the major changes in hormone contents occurred at 15 h, immunocytolocalization has been performed at this time point in our three conditions. As showed in Fig. 5, ABA was observed mainly in the nucleus in all sections of D 10 °C and in the cytosol for D 20 °C and ND both in the axis and the meristematic zone (ABA 1 and 2, respectively, Fig. 5e). ABI5 was observed mainly in the nucleus in all sections of D 10 °C (as shown for the axis in the Fig. 5e) and in both of the nucleus and the cytosol for D 20 °C, however, less fluorescence has been observed in the nucleus. In ND 10 °C, its major localization was observed in the cytoplasm (Fig. 5e). RGL2 exhibits similar pattern of localization as for ABI5 but with larger proportion of labelled nuclei in D 20 °C comparable to that of D 10 °C (Fig. 5e). However, nucleus/cytoplasm fluorescence ratio using ImageJ quantification showed the importance of the fluorescence in the cytoplasm in D 20 °C as compared to D 10 °C (9,43 and 1,93, respectively). ACO, unlike ABI5 and RGL2, was localized in the cytoplasm in all conditions. Fluorescence showed comparable patterns in the axis (Fig. 5e, ACO 1), but interestingly, it was almost undetected in the meristem of D 10 °C and significantly increase in D20 °C and ND 10 °C (Fig. 5e, ACO 2). This finding was confirmed with fluorescence quantification (Supplemental Fig. 2). Labelling in the cotyledon parts (3 in Fig. 5a) was comparable to that of the axis for all antibodies (Supplemental Fig. 3).

## Discussion

Increase in temperature and after-ripening are known to allow seed dormancy release in sunflower. To study the determinism of these processes, we analysed in sunflower seeds the ABA, GAs and ethylene contents, the expression of genes involved in their synthesis or signalisation and the localization of some of their effectors in dormant conditions (D 10 °C) and dormancy breaking conditions by temperature (D 20 °C) or after ripening (ND). A decrease in ABA content was observed in D 10 °C and ND 10 °C sunflower seeds after 15 h of imbibition. After 24 h, the ABA content re-increased in D 10 °C but not in ND 10 °C. These data are in accordance with the well-known role of ABA in maintenance of dormancy since the endogenous ABA content was shown to decline upon imbibition in ND and D seeds during the early phase of germination, this decrease continuing in ND seeds while subsequent *de novo* ABA synthesis occurred in imbibed D seeds leading to dormancy maintenance^10,53–57^. The higher expression of NCEDs (*HaNCED2* and *HaNCED4)* key enzymes of the ABA biosynthesis^24,58–60^ could explain the increase in ABA content for non-germinating dormant D 10 °C seeds and germinating D 20 °C seeds. *HaNCED4* was induced more importantly in D 20 °C compared to D 10 °C at 3 h what is in agreement with the *LsNCED4* expression required for thermoinhibition of lettuce seeds as it plays a role in plant response to elevated temperature^61^. *HaABA2*, which encodes an enzyme catalyzing the last two steps in ABA biosynthesis, was also up-regulated in D 20 °C in accordance with the increased level of ABA in these seeds. On the opposite, the expression of *HaNCED2* was reduced in ND seeds at 10 °C when compared to D 10 °C and D 20 °C seeds. The concomitant larger increase expression in ND at 6 and 15 h of *HaCYP707A1*, a gene that putatively encodes an enzyme catalyzing ABA hydroxylation may contribute importantly to ABA degradation^24,58–60^, which can explain the decrease in ABA content recorded in ND seeds. These data are in accordance with previous studies showing that down regulation of *HvABA8*′*OH-1* expression and up-regulation of *HvNCED1* and *HvNCED2* are correlated with the secondary dormancy due to high temperature^62^ and those showing that *HvNCED1* and *HvNCED2* together with *HvABA8*′*OH-1*, play key role in endogenous ABA regulation at transcriptional level in barley^63,64^. The different gene regulations observed between D 20 °C and ND 10 °C seeds are in accordance with the measured ABA contents and indicate that ABA content is regulated in different ways during temperature and after-ripening dormancy alleviation. Indeed, ABA content increased in D 20 °C during imbibition similarly to D 10 °C.

These results suggest that ABA effect is counteracted in germinating D 20 °C although its endogenous level remained high, when after-ripening could be associated with the ABA content decrease. Cytological study brings new information concerning ABA localization difference between germinating and non-germinating seeds. Indeed, ABA localized mainly in the nucleus in non-germinating seeds (D 10 °C), while it localized in the cytoplasm in germinating seeds. Nucleus ABA localization has never been reported but it is consistent with localization of its receptors PYR1 reported in nucleus and cytoplasm^65,66^. PYR/PYLs were shown to play a major role in germination regulation^67^. Furthermore, it was demonstrated that nuclear PYR1 is sufficient to generate ABA responses, such as the inhibition of seed germination^68^.

Genetic and molecular analysis revealed many important regulators of ABA signalling. The major ABA signalling for ABA-dependent gene expression including AREB/ABF regulon, SnRK2 protein kinase, group A (or clade A), 2C-type protein phosphatases (PP2Cs) and ABA receptors have been proposed^69^. However, in our study, no significant difference between samples has been observed for ABA signalling genes suggesting that their regulation may take place at the post-transcriptional or -translational level. It was the case for the plant basic leucine zipper (bZIP) transcription factor ABI5 known to be a key regulator in the ABA signalling pathway controlling seed dormancy and germination^70^. ABI5, as a responsive transcription factor, is localized in nucleus and mainly expressed in the seed germination stage^70^, but it can shuttle between the nucleus and the cytosol, a cytoplasmic degradation preventing nuclear accumulation of ABI5 and prohibiting ABA signalling^71^. Accordingly, our cytological analysis showed that ABI5 is present in all the tested conditions but localized only in the nucleus for D 10 °C while it is localized mainly in the cytoplasm for ND 10 °C and in both compartments for D 20 °C. ABI5 could be degraded in the cytoplasm in germinating, D 20 °C and ND 10 °C, seeds probably by oxidation and/or ubiquitination^72,73^. Thus, in sunflower seeds, ABI5 does not seem to be regulated at the transcriptional level but at the post-translational level. Exclusion of ABI5 from the nucleus and its degradation could explain the decrease in sensitivity to ABA observed in ND 10 °C seeds independently of their endogenous level.

The antagonistic function between ABA and GAs in seeds has been extensively reviewed^36,37,74–76^. We effectively could record in our model larger increases in GA_1_ content in germinating D 20 °C and ND 10 °C seeds when compared to non-germinating D 10 °C seeds. The gene expression regulation of GA3ox and GA2ox, involved in GAs synthesis and degradation, respectively, at 3 h for D 20 °C and at 15 h for ND when compared to D 10 °C, are in accordance with a larger synthesis than a degradation, explaining thus the GA_1_ increase observed at 15 and 24 h for D 20 °C and ND 10 °C, respectively. Thus, increase in temperature like after-ripening treatment induced GAs biosynthesis lowering the ABA/GAs ratio and allowing germination. It is further noteworthy that the localization of RGL2, a key DELLA factor repressing germination^30^, targets in the same cellular compartments as ABI5 under the different conditions tested. These data could be related to previous studies showing that GAs promotes seed germination by enhancing the proteasome-mediated destruction of RGL2 in the cytosol and that RGL2 inhibits seed germination by stimulating ABA synthesis and ABI5 activity in Arabidopsis suggesting that ABI5 acts as the final common repressor of germination in response to changes in ABA and GAs levels^51^. Experimental evidence has shown that a decrease in endogenous GAs content leads to an increase in ABA synthesis through a stabilized RGL2, meanwhile, the transcript and protein levels of both of ABI5 and RGL2 are induced by an increased endogenous ABA content^51,77^. Thus, in addition to the increase in GAs content favoring the decrease in the ABA/GAs ratio, the exclusion of RGL2 from the nucleus should allow an increase in sensitivity to GAs in D 20 °C seeds and ND 10 °C seeds favoring also dormancy alleviation (Fig. 1).

From these data it seems that the intracellular cytoplasmic relocalizations of ABI5 and RGL2 could allow their degradation and thus favor the GAs effect and de-favor the ABA effect leading to germination. Concerning ACO it seems that a tissue relocalization in the meristem is necessary to favor ethylene effect and germination.

Ethylene was also shown to alleviate dormancy and promote germination in sunflower seeds^5,32^ and many other species^32^. In this study, we showed that ethylene was not detected in the different conditions. However, the gene expression of the key ethylene biosynthesis enzyme, ACO was induced at 3 h in D 20 °C. Furthermore, *HaACO* expression was increased later in ND 10 °C when compared to D 10 °C seeds, which suggests that ethylene biosynthesis could also be stimulated to improve seed dormancy alleviation in ND seeds but that the amount of produced ethylene could be too low to be detected. In fact, ethylene has been detected concomitantly with radicle protrusion in ND sunflower seeds^78^. Ethylene production was effectively already associated with after-ripening in several species^79–81^ and it has been proposed that endosperm cap weakening and rupture are promoted by ethylene in *Lepidium*^56^. Our data show that ACO is present in the cytoplasm for all the three conditions which is in accordance with ACO localization in the cytoplasm that has been reported in cultured tomato cells, apple fruit pericarp or phloem cells^82–84^ it is noteworthy that ACO have not been be detected in the meristematic zone in non-germinating seeds (D 10 °C) when it was present in the meristem of germinating seeds (D 20 °C and ND 10 °C). These data suggest the need of a specific spatial distribution of ACO in the seed for germination achievement. Tissue localization of ACO, the last enzyme in the ethylene biosynthetic pathway, was proposed to be relevant for ethylene action or diffusion^85^. In this study, we suggest that ethylene production could be localized in the meristem which represents the growing zone. Ethylene could thus be a prerequisite and not only a consequence of germination promotion.

**Figure 6:**
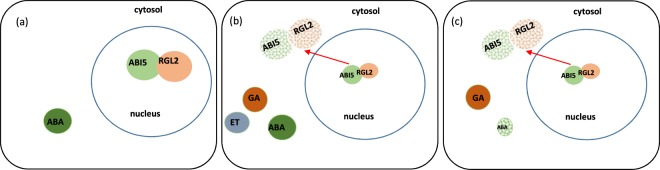
Scheme representing determinant mechanisms of hormonal regulation in seed dormancy alleviation. (**a**) dormant seed cell incubated at 10 °C characterised by ABA presence and its signalling action by ABI5. (**b**) Dormant seed cell incubated at 20 °C characterised by the degradation of ABI5 and RGL2, the increase of GA and ethylene (ET) in the presence of ABA (**c**) non dormant seed cell at 10 °C characterised by the degradation of ABI5 and RGL2, the increase of GA and ET and the decrease of ABA content.

As a whole our data showed that after-ripening mediated release of seed dormancy followed the classically described process involving a decline in ABA content and an increased GAs content. However, we showed that decreased ABA content is not a prerequisite for temperature-induced germination in sunflower but that increases in GAs and potentially ethylene are sufficient to counteract the presence of ABA if ABI5 re-localization and degradation allow decreasing the sensitivity to ABA. Effectively, temperature- and after-ripening-induced dormancy release seemed dependent mostly on the re-localization of ABI5 and RGL2 in the cytoplasm allowing sensitivity change to ABA and GAs and the localization of ACO in the meristematic zone to allow on-site ethylene production (Fig. 6). Thus the regulation of the sensitivity to hormones by re-localization of their effectors appeared as an important issue explaining the possible dormancy alleviation with various hormones contents.

## Supplementary information

supplemental figures
