# Supplementary material for: Re-localization of hormone effectors is associated with dormancy alleviation by temperature and after-ripening in sunflower seeds
Source: Sci Rep. 2019 Mar 19;9:4861. doi: 10.1038/s41598-019-40494-w (PMC6424972; doi:10.1038/s41598-019-40494-w)
Supplement: Supplementary file 1 — supplemental figures [file 41598_2019_40494_MOESM1_ESM.pdf]

Re-localization of hormone effectors is associated with dormancy alleviation by temperature and after-ripening in sunflower seeds

Qiong Xia[1], Maharajah Ponnaiah[1], Kaviya Thanikathan Subramanian[1], Françoise Corbineau[1], Christophe Bailly[1], Eiji Nambara[2], Patrice Meimoun[1§] & Hayat El-Maarouf-Bouteau[1§*]

[1]*Sorbonne Université, CNRS, Biologie du développement Paris Seine - Institut de Biologie Paris Seine, LBD - IBPS, 75005 Paris, France.*
[2]*Department of Cell and Systems Biology, University of Toronto, Toronto, ON M5S 3B2, Canada.*

§ participate equally to the work
*Corresponding author
E-mail: hayat.bouteau@upmc.fr (HEMB)

Table S1.

Sequences of primers used for qRT-PCR experiments. Heliagene accession number for each gene was determined by https://www.heliagene.org and the sequence of primer by Primer6. The *HaACO* corresponding primers were from [44] and the internal standard gene primers, EFR, Tubulin and S19, were from [45].

Name of H. annnus L. gene	Heliagene Accession number	Homology percentage of EST sequences with other plants (plant; accession number)	Amplification product size(bp)	Primer sequences
HaNCED2	Ha412v1r1_15g037960	80 (Lactuca sativa; AB120108)	147	F: TGGAGACGCCGACTATGGTTCA<br>R: CGCCGAAACGTGACACCTTCT
HaNCED4	Ha412v1r1_08g038660	99 (Helianthus annuus;LN871186)	110	F: TGCGAAAGACGAGTCCGAGATG<br>R: GAGCCACCATCACCACCGTATC
HaCYP707A1	Ha412v1r1_17g006190	76 (Arabidopsis thaliana; NM_118043)	181	F: GATTCGCTTCGGTCGTTGGAA<br>R: CGGGAAGGTTAATCGGCATCG
HaCYP707A2	Ha412v1r1_05g007770	70 (Arabidopsis thaliana; NM_001336198)	84	F: GCAAAGATGGTGTTGGTGAGTG<br>R: GGCTTCTGGGCCGATCATCT
HaCYP707A3	Ha412v1r1_06g014700	74 (Arabidopsis thaliana; NM_180805)	161	F: CGATGAAGGCGAGGAAAGAGC<br>R: GCAGCGAATATGACACCGATGA
HaHAB1	Ha412v1r1_09g045480	79 (Cirsiumarvense; JQ599224)	88	F: TGTTGAGGACGTGCCGATTGA<br>R: TTCCGCCATTACAGACGCCTT
HaHAB2	Ha412v1r1_10g062350	73 (Solanum lycopersicum; NM_001247757)	85	F: CCATTCCCAAGGAACTCTCTCG<br>R: AGCTTCCTCTTTCTTGGCAACA
HaABI2	Ha412v1r1_09g001950	74 (Morusnotabilis; XM_010097045)	86	F: GGAGGATGTACCGCCGGAAGTT<br>R: GAACCGCCATGACCGTCGTAA
HaSnRK2s-1	Ha412v1r1_15g036900	80 (Nicotianatabacum; XM_016614247)	119	F: ACAGTCGGTACACCAGCATACA<br>R: TAGCCGCCAATGAGCATGATGT
HaSnRK2s-2	Ha412v1r1_12g044140	75 (Medicago sativa; JX187639)	135	F: CGTCATGCTTGTTGGTGGCTAT<br>R: AGGTGGCGACATTCAGGTGAT
HaABI3	Ha412v1r1_15g006390	78 (Arabidopsis thaliana; NM_113376)	93	F: TGGAGGGAACTGGGTGTACTGG<br>R: AACGGTCACAGGACGGAACAG
HaABI5	Ha412v1r1_00g108760	75 (Arabidopsis thaliana; NM_001336591)	148	F: CACCAACAGCCAAGCAATGACA<br>R: CAGGCGGAGCATTCGGATGAT
HaGA3ox1	Ha412v1r1_10g044740	87 (Lactuca sativa; AB636282)	93	F: TCCTGATCCTGACCGAGCCATG<br>R: GCCTGGAGTCCACTTGTGTTGT
HaGA2ox	Ha412v1r1_10g051610	81 (Lactuca sativa; AB031206)	75	F: ACTTTGGAGGACCACCATTGAA<br>R: ACAAGCTGTCTTCTTCCCTTTC

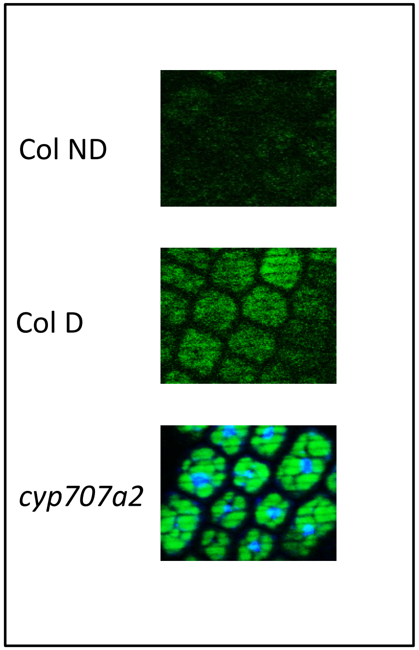

Figure S1.
ABA immunocytolocalization showing ABA accumulation in *cyp707a2* mutant comparing to Columbia (col) dormant (D) and non-dormant (ND) seeds.

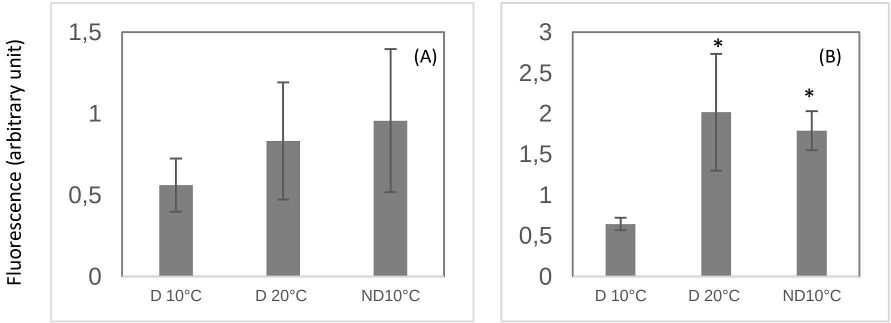

Figure S2.
Fluorescence quantification of ACO labelling using ImageJ software. **(A)** quantification in the tip of the axis (zone 1), **(B)** quantification in the meristematic zone (zone 2).

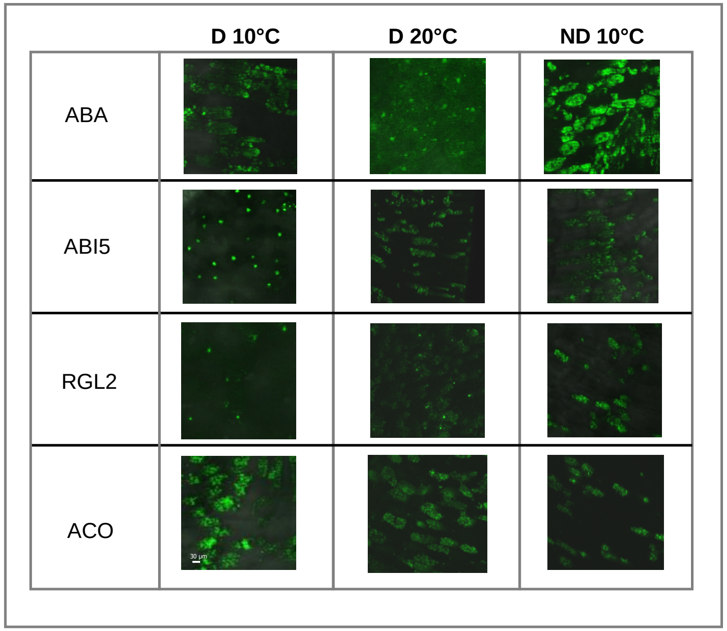

Figure S3.

Immunolocalization of ABA, ABI5, RGL2 and ACO in cotyledons of sunflower embryos. Longitudinal sections were prepared from naked seeds imbibed after 15 h on water. Green label indicates the interaction of different antibodies with their antigen.
